# Bayesian probability estimates are not necessary to make choices satisfying Bayes’ rule in elementary situations

**DOI:** 10.3389/fpsyg.2015.01194

**Published:** 2015-08-17

**Authors:** Artur Domurat, Olga Kowalczuk, Katarzyna Idzikowska, Zuzanna Borzymowska, Marta Nowak-Przygodzka

**Affiliations:** ^1^Department of Cognitive Psychology, Faculty of Psychology, University of WarsawWarsaw, Poland; ^2^Center for Complex Systems and New Technologies, The Robert B. Zajonc Institute for Social Studies, University of WarsawWarsaw, Poland; ^3^Centre for Economic Psychology and Decision Sciences, Kozminski UniversityWarsaw, Poland; ^4^Laboratory of Visual System, Nencki Institute of Experimental BiologyWarsaw, Poland

**Keywords:** Bayes’ rule, choices, binary hypothesis, heuristics, natural sampling, ecological rationality, non-inverse rule

## Abstract

This paper has two aims. First, we investigate how often people make choices conforming to Bayes’ rule when natural sampling is applied. Second, we show that using Bayes’ rule is not necessary to make choices satisfying Bayes’ rule. Simpler methods, even fallacious heuristics, might prescribe correct choices reasonably often under specific circumstances. We considered elementary situations with binary sets of hypotheses and data. We adopted an ecological approach and prepared two-stage computer tasks resembling natural sampling. Probabilistic relations were inferred from a set of pictures, followed by a choice which was made to maximize the chance of a preferred outcome. Use of Bayes’ rule was deduced indirectly from choices. Study 1 used a stratified sample of *N* = 60 participants equally distributed with regard to gender and type of education (humanities vs. pure sciences). Choices satisfying Bayes’ rule were dominant. To investigate ways of making choices more directly, we replicated Study 1, adding a task with a verbal report. In Study 2 (*N* = 76) choices conforming to Bayes’ rule dominated again. However, the verbal reports revealed use of a new, non-inverse rule, which always renders correct choices, but is easier than Bayes’ rule to apply. It does not require inversion of conditions [transforming P(H) and P(D|H) into P(H|D)] when computing chances. Study 3 examined the efficiency of three fallacious heuristics (pre-Bayesian, representativeness, and evidence-only) in producing choices concordant with Bayes’ rule. Computer-simulated scenarios revealed that the heuristics produced correct choices reasonably often under specific base rates and likelihood ratios. Summing up we conclude that natural sampling results in most choices conforming to Bayes’ rule. However, people tend to replace Bayes’ rule with simpler methods, and even use of fallacious heuristics may be satisfactorily efficient.

## Introduction

This paper aims to investigate whether people conform to Bayes’ rule when making choices in probabilistic situations, or whether they tend to simplify their reasoning by using other methods. To develop an understanding of what a Bayesian problem is, consider the following example:

***The red nose problem*** (Zhu and Gigerenzer, 2006, p. 289).

Pingping goes to a small village to ask for directions. In this village, 10 out of every 100 people will lie. Of the 10 people who lie, eight have a red nose. Of the remaining 90 people who do not lie, nine also have a red nose. Imagine that Pingping meets a group of people in the village with a red nose. How many of these people will lie?

The red nose problem illustrates an elementary situation, which is defined with binary sets of hypotheses (H and not-H) and data (D and not-D). A person can lie or not and have a red or non-red nose. There can be several hypotheses and data sets, but discussing such situations is beyond scope of the present article. According to Bayes’ rule, an estimate of the posterior probability of a distinct hypothesis should be computed using the observations provided and the prior probability of the hypothesis. In the example, the goal is to compute the posterior probability of being a liar given that a person has a red nose. We denote it with P(H|D) and compute it with the formula:

(1) P(H|D)=P(HandD)/P(D)=P(H)P(D|H)/[P(H)P(D|H)+P(not-H)P(D|not-H)]

To perform the calculation we need to know the base rate P(H), which is the chance of a person being a liar, P(H) = 10/100 = 10%. Thus, the chance of being a non-liar, P(not-H) is 90%. This should be updated with new data, conditional probabilities P(D|H) and P(D|not-H). Hence, there are P(D|H) = 8/10 = 80% of people with a red nose among liars, and P(D|not-H) = 9/90 = 10% among non-liars (people who have a red nose when they tell the truth). This enables computation of whether a person with a red nose will lie:

P(H|D)= 10%×80%/[10%×80%+ 90%×10%]=47%

The conclusion is that those with a red nose will lie with a 47% probability.

Bayesian estimates are counter-intuitive and people are usually surprised by the discrepancy among a base rate (10% in the above example), likelihood ratio (80%), and actual Bayesian probability (47%). Similar discrepancies occur in such well-known cases as the taxi cab problem (Tversky and Kahneman, 1982) and the mammography problem (Eddy, 1982). In the taxi cab problem, given a witness’s evidence that a cab was blue, the probability that the cab was actually blue is 41%. This Bayesian result differs from – specified in the case – the base rate of 15% and the likelihood ratio of proper color identification which equals 80%. In the mammography problem, while the base rate of breast cancer is 1% and the likelihood ratios are 80% for a positive test and 9.6% for a false alarm, the actual Bayesian probability is 7.8%.

Numerous studies show that people have difficulty in finding solutions for Bayesian problems. Subjects acquainted with new evidence are conservative and underestimate posterior chances (Phillips and Edwards, 1966; Edwards, 1968). They also demonstrate the base rate fallacy, neglecting P(H), and the inverse fallacy, confusing likelihood ratios P(D|H) with Bayesian estimates P(H|D) (Koehler, 1996; Villejoubert and Mandel, 2002). Systematic ignorance of prior probabilities and overuse of the representativeness heuristic have led to the conclusion that people are not Bayesians (Kahneman and Tversky, 1972, 1973; Tversky and Kahneman, 1982).

Misapprehension of the probabilities may lead to inadequate decisions and entail severe consequences. Gigerenzer et al. (1998) reported the case of seven out of 22 blood donors who committed suicide after they were shown to be HIV-positive by the ELISA and Western Blot tests, which had a 100% detection efficiency. It transpired that the actual Bayesian probabilities were around 50%. The authors concluded that there is a need to develop tools for understanding and appropriately communicating risks in AIDS counseling centers. Such problems occur not only in the domain of medical diagnosis but in other domains where probabilistic evaluations depend on both prior distributions and newly obtained information (e.g., in management, law and intelligence analysis – see Nance and Morris, 2005; Hoffrage et al., 2015; Mandel, 2015). A vast amount of research was focused on pedagogical issues surrounding Bayesian inference. Methods were elaborated to aid the understanding of Bayes’ rule and facilitate communication of risk appropriately. These used visual representations such as Venn diagrams, trees, pictorial representations, or frequency grids (Mellers and McGraw, 1999; Yamagishi, 2003; Brase, 2008; Mandel, 2014; Navarrete et al., 2014; Sirota et al., 2014).

Bayesian reasoning issues have been of particular interest to evolutionary psychologists, who have proposed an ecological rationality framework for research (Gigerenzer and Hoffrage, 1995; Cosmides and Tooby, 1996; Brase et al., 1998). According to this approach, people are not evolutionarily prepared for performing abstract computations. In particular, the concept of probability is an ecologically invalid notion. The calculus of probability is a relatively recent discovery in humankind’s history, and the human mind having evolved to maintain information in the form of absolute numbers. Such numbers are termed natural frequencies and the process of gathering information on natural frequencies through real life experience is termed natural sampling (Kleiter, 1994; Gigerenzer and Hoffrage, 1995; Gigerenzer, 1998). Because humans have collected information in the form of natural frequencies throughout evolution, such representations facilitate correct Bayesian reasoning (Cosmides and Tooby, 1996; Sedlmeier and Gigerenzer, 2001).

For example, the natural frequencies in the red nose problem are:

• The total number of village inhabitants, *a* = 100,
• Numbers of liars, *b* = 10,
• Non-liars, *c* = 90,
• People with a red nose among liars, *d* = 8,
• Liars with no red nose, *e* = 2,
• Non-liars with a red nose, *f* = 9,
• Non-liars with no red nose, *g* = 81.

Studies by Zhu and Gigerenzer (2006) showed that even children can give appropriate answers to Bayesian problems if they are presented with natural frequencies. The frequencies simplify computations because posterior probabilities can be estimated as:

P(H|D)=d/[d+f]

To compute whether a person with a red nose will lie it is sufficient to calculate:

P(H|D)=8/(8+9)=47%

The evolutionary approach has been criticized for being difficult to falsify (Girotto and Gonzalez, 2001). While people deal with natural formats better than with probabilities, this does not necessarily mean that this ability has developed through natural selection or adaptation. One cannot simply rely on previous experience to perform successfully in a novel or complex environment. Frequencies help visualize nested sets and relations, and thereby facilitate solution of Bayesian problems, but this does not necessarily result from Bayesian inference (Sloman et al., 2003). Solving probabilistic problems requires also the comprehension of elementary logic, set operations and relations (Barbey and Sloman, 2007). For instance in Girotto and Gonzalez (2001) studies subjects performed better when subset relations were activated.

We agree that the evolutionary approach is not convincing in its explanation of how reasoning developed, and the issue of how the ability to collect and process natural frequencies developed in humans is debatable. However, there is agreement that natural frequencies are easier to process and that people learn about statistical relationships from natural sampling in real life. Hence, the ecological framework seems to be valid at least in that:

(1) Statistical information is gathered via natural sampling,
(2) The environment defines objectives and supplies means to achieve them, and
(3) Human rationality is ecological.

Nevertheless, these propositions lead us to conclude that single probability judgments do not provide sufficient information for attaining goals in situations such as the red nose problem, where choices are placed before people in fact. In the original story (Zhu and Gigerenzer, 2006), Pingping’s goal is to obtain the right directions to continue his journey, and he is expected to assess the chance of being cheated by people with a red nose. However, exploring the truthfulness of people with red noses only is not enough: Pingping has to decide whether to ask for directions someone with a red nose or refrain from this and ask a person without a red nose, actually. ‘Having no red nose’ is also a clue with some ecological validity. Thus, we propose a modified question in the red nose problem:

Should Pingping ask a person with a red nose for directions, or find a person who does not have a red nose?

What works for evaluating the truthfulness of people with red noses will also work for evaluating the truthfulness of people without red noses. To answer the question, Pingping should calculate the proportion of liars among both people with red-noses and people without red noses, applying Bayes’ rule twice:

• P(H|D) = *d*/(*d* + *f*) = 8/(8 + 9) = 47%
• P(H|not-D) = *e*/(*e* + *g*) = 2/(2 + 81) = 2%

Having compared these chances, Pingping should conclude that he takes a far greater risk of being lied to when he asks someone with a red nose and conclude that it is better to find someone without a red nose.

Reconsidering the red nose problem in such a way shows that, to solve such problems, estimates referring to all the options are needed. This is in the line with probabilistic functionalism, which proposes that people do not evaluate probabilities for their own sake, but to achieve specific goals. People infer missing data from probabilistic indicators to reduce incompleteness and uncertainty in their knowledge (Brunswik, 1943; Dhami et al., 2004; Pleskac and Hertwig, 2014).

There is common agreement that natural sampling may facilitate correct Bayesian reasoning. People acquire knowledge about probabilities from their own experience rather than compiled frequency statistics (Gigerenzer, 1998). Surprisingly, natural sampling is not reflected in most experiments, where participants are provided with well-prepared and well-arranged natural frequencies or probabilities (Kleiter, 1994; Girotto and Gonzalez, 2001). We postulate that experiments should attempt to approximate the experiential aspect of natural sampling. However, such experiments should not give clues to participants about processing data at the same time. An understanding of conditions in general is a crucial step in solving a Bayesian problem. After realizing that the inferential process should be narrowed to a given condition (the first step in Eq. 1), one should invert one’s thinking about conditions from *D*|*H* into *H*|*D* (the second part of Eq. 1). Framing tasks with natural frequencies (“Imagine that Pingping meets a group of people in the village with red noses. How many of these people will lie? __ out of __,” as originally in Zhu and Gigerenzer, 2006, p. 289) is suggestive and entails scaffolding the answers. The group characterized by data D is identified directly (“these people”) and the subsequent question suggests narrowing thinking to this set (“__ out of __”). A person has no need to perform the first step on their own in tasks framed this way, and the clue about how to answer helps people to avoid comitting the inverse fallacy. Hence, we postulate that research techniques should reflect natural sampling, but in a way that gives no clues to participants about how to process probabilistic information.

In our studies, we mimic the process of natural sampling and present participants with actual events instead of probabilities or frequencies. We anticipate that participants should have learned these from their own experience and that they should make choices based upon them. This approach reflects a paradigm in which decisions based upon participants’ own experience are explored, as proposed by Hertwig et al. (2004) and continued by their followers (for a review, see Hertwig and Erev, 2009; Rakow and Newell, 2010). As these researchers argue, people make everyday decisions, such as backing-up a hard drive or crossing a busy street, by relying on the recall of events that they have previously experienced, not based upon descriptions of outcomes or likelihoods (Hertwig et al., 2004). Everyday decisions or choices rarely need to be articulated in exact numbers and the outcome of one’s inference is usually expressed in his actions or choices, not in estimates of probabilities. Therefore, it should be easier for people to deal with Bayesian problems by choosing between two alternatives (differing with respect to a posteriori probabilities of success) rather than giving exact numbers. Hence the first question regarding choices in elementary situations that we aim to answer is:

[Q1] How often do people make choices satisfying Bayes’ rule, when probability information is gathered through natural sampling?

In answering Q1, one can expect that [H] choices will conform to Bayes’ rule in natural sampling settings:

[Ha] in most of the tasks (a strong criterion) or

[Hb] more frequently than at random (a weak criterion).

On the one hand, people tend to maximize their performance. It should also be easier for people to articulate a solution by choosing between two alternatives, rather than articulating exact numbers. Hence, their choices should comply with Bayesian rule (Ha). On the other hand, using the rule is cognitively costly, so it may often be ignored or replaced with heuristics or other methods. For instance, comparing fractions may turn out to be just as hard as comparing probabilities or percentages (Kleiter, 1994; Gigerenzer and Hoffrage, 1995; Gigerenzer, 1998). Even if fractions are estimated properly, computational complexity increases with the necessity of performing two correct Bayesian evaluations and performing a correct comparison of them when making choices. We therefore also formulated a weaker expectation that the choices would comply with Bayes’ rule more frequently than other methods (Hb).

To answer question Q1, we created experiments that reflected natural sampling, with the intention of showing how often people make choices satisfying Bayes’ rule (Studies 1 and 2).

Using Bayes’ rule requires cognitive effort and only pays-off when one can make significantly better decisions. Cognitive limitations and the avoidance of effort make people turn to the use of fallacious heuristics, which are popular because they are frugal and still roughly correct (Gigerenzer et al., 1999; Gigerenzer, 2004, 2008). As Simon (1955, 1956) hypothesized, people select strategies that meet minimal standards and aspirations. Ecological rationality postulates that calculations do not have to be correct, however, they should be correct reasonably often (Gigerenzer, 2004; Over, 2004). As Gigerenzer (2008, p. 25) further explained, “The goal of an organism is not to follow logic, but to pursue objectives in its environment, such as establishing alliances, finding a mate, and protecting offspring. Logic may or may not be of help. The rationality of the adaptive toolbox is not logical, but *ecological*; it is defined by correspondence rather than coherence.”

Summing up, the interesting issue is whether heuristics can prescribe correct answers satisfactorily often, given some specific circumstances. Thus, we raise the question:

[Q2] How often do fallacious heuristics yield choices that conform to Bayes’ rule?

Zhu and Gigerenzer (2006) observed that, instead of Bayes’ rule, people use the following fallacious heuristics (following these authors, we apply the term “cognitive strategies” or in short “strategies” describing them and Bayes’ rule):

• the conservatism strategy: *b*/*a*,
• the evidence-only strategy: (*d* + *f*)/*a*,
• the representativeness strategy: *d*/*b*,
• the pre-Bayesian strategy: *b*/(*d* + *f*).

By analogy, people may apply these cognitive strategies to simplify their choices in elementary situations through the following comparisons:

• the evidence-only strategy: comparing (*d* + *f*)/a with (*e* + *g*)/*a*,
• the representativeness strategy: *d*/*b* with *e*/*b*,
• the pre-Bayesian strategy: *b*/(*d* + *f*) with *b*/(*e* + *g*), and
• the conservatism strategy: *b*/*a* with *c*/*a*.

In the red nose problem, the Bayes’ rule, the representativeness strategy (*d*/*b* = 8/10 > *e*/*b* = 2/10) and the pre-Bayesian strategy [10/(8 + 9) > 10/(2 + 81)] would result in a decision not to ask a person with a red nose. Only the evidence-only strategy would render a different conclusion [(8 + 9)/100 < (2 + 81)/100].

To answer question Q2 we investigated how often fallacious strategies (representativeness, pre-Bayes, and evidence-only) prescribe the same choices as Bayes’ rule by carrying out computer simulations of natural frequencies (Study 3).

## Study 1

The goal of Study 1 was to answer Q1: how often do choices conform to Bayes’ rule in elementary situations?

### Materials and Methods

We used a computer program with a sequence of 16 simulation tasks, which we called “adventures.” Introductory instructions were as follows: “The study you will be taking part in is aimed at finding out how people find precious objects. You will be presented with 16 opportunities to acquire precious objects: diamonds and amber. Each of the 16 adventures consists of two stages. The initial phase should familiarize you with the area. The second part requires you to identify where the gem is hidden. Each adventure is independent and concerns treasures in the form of diamonds or amber. The next screen will reveal the first phase of adventure number one. You will be presented with seven cards. On the face of each card you will find a diamond (a piece of amber) or a stone (a piece of broken glass). Clicking the card will turn it over and reveal a color: green or yellow. Your task is to click on, i.e., turn over, all the cards to reveal colors on the back of the diamonds and stones. In the second stage, you should select the card with the color that has a diamond or a piece of amber underneath. You will take part in 16 such adventures.” Subsequently, participants were asked if they understood the instructions. If so, they proceeded to perform the 16 tasks. Half of the adventures contained diamonds and stones, the other half, amber and glass, respectively. (For clarity, henceforth we only describe the method referring to diamonds and stones.)

Each adventure consisted of two stages.

**FIGURE 1:**
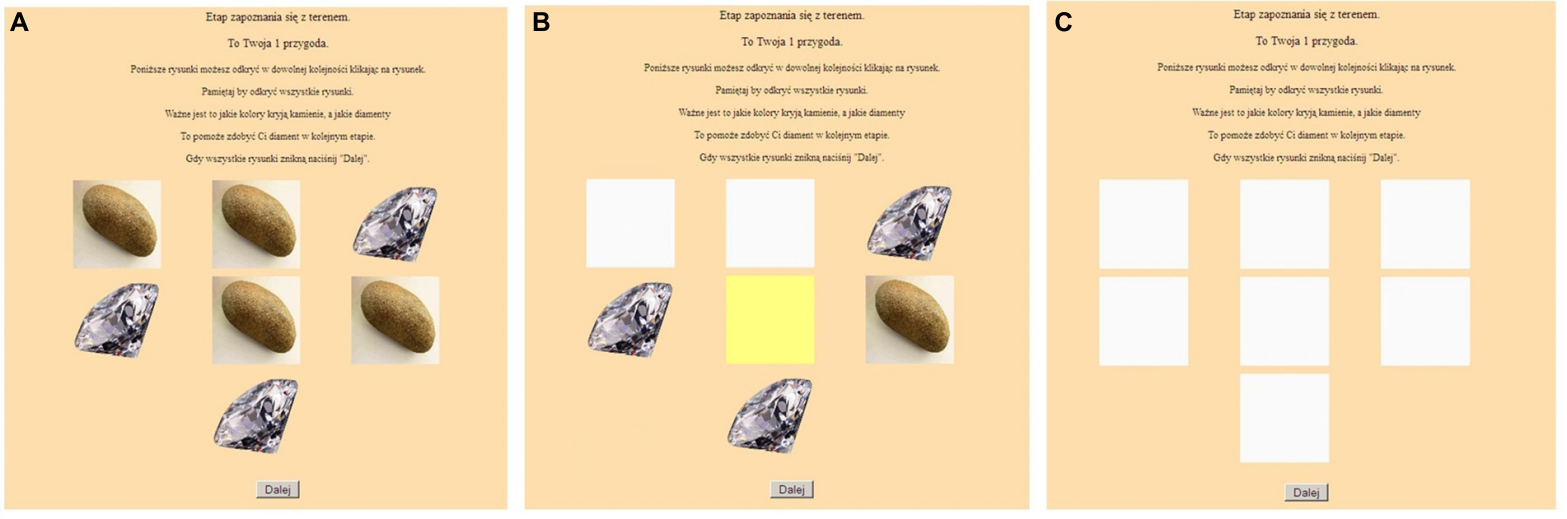
**The computer task: the learning stage – **(A)** before, **(B)** during, and **(C)** after turning over the cards**.

The first stage was the learning stage, which was a simulation of natural sampling and was intended to develop intuition about Bayesian relationships. A participant was presented with seven cards showing valuable objects or worthless items on their faces (**Figure 1A**). The participant was instructed to turn over all of the cards in order to reveal the colors on their backs (**Figures 1B,C**). The person was to remember the colors associated with diamonds and stones, which would help them to acquire a diamond in the next step. Yellow and green colors were used for the back of the cards because these colors have relatively neutral emotional connotations (Karp and Karp, 1988).

In probability terms, a participant could learn:

• Prior occurrences of diamonds [*b*/(*b* + *c*)] and stones [*c*/(*b* + *c*)];
• Likelihood ratios for the backs of diamonds: green [*d*/(*d* + *e*)], yellow [*e*/(*d* + *e*)];
• Likelihood ratios for the backs of stones: green [*f*/(*f* + *g*)], yellow [*g*/(*f* + *g*)];
• Bayesian estimates of revealing a diamond for backs: green [*d*/(*d* + *f*)], yellow [*e*/(*e* + *g*)].

**FIGURE 2:**
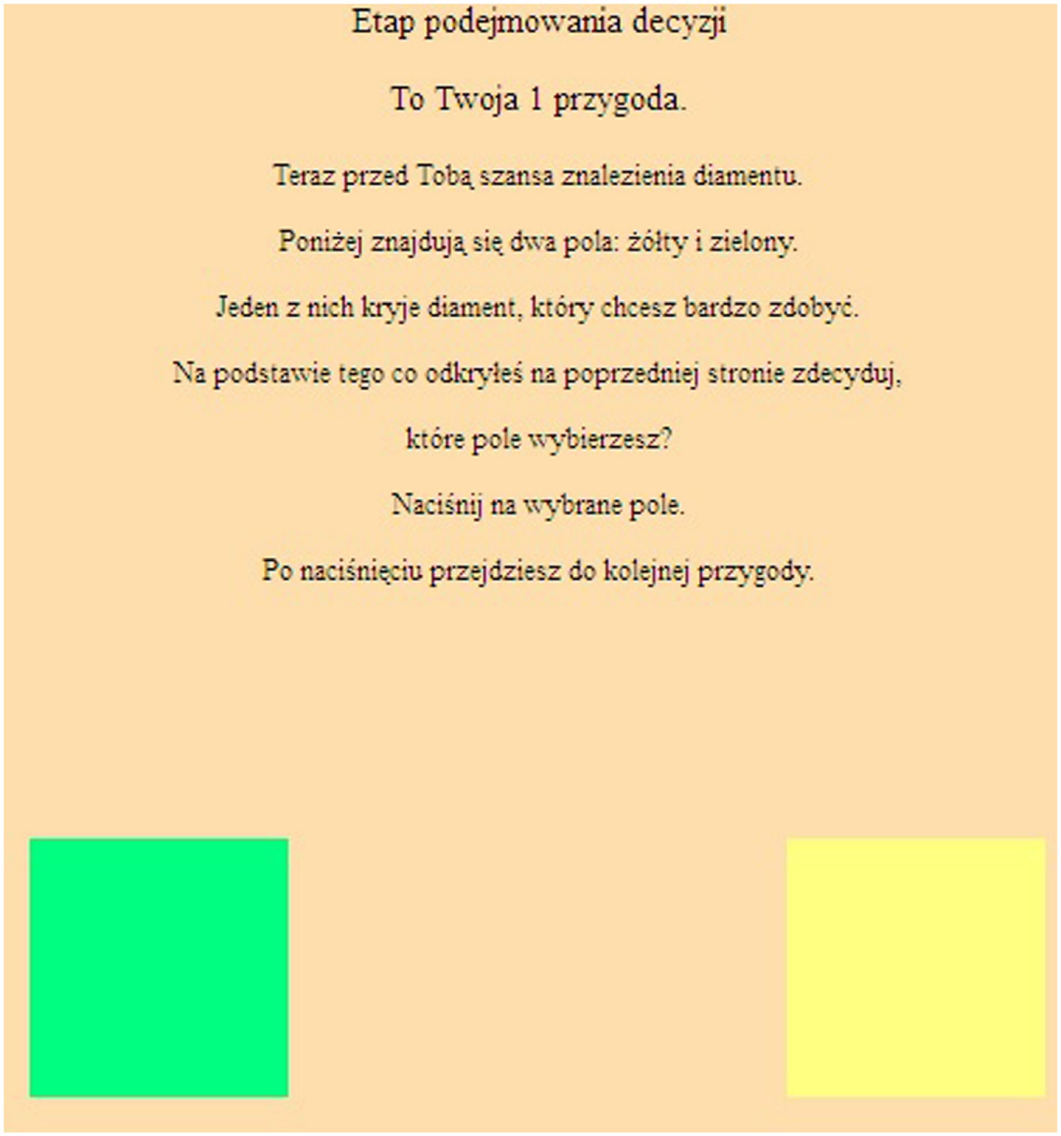
**The computer task: the color choice stage**.

In the second stage of the adventure, participants chose between two differently colored cards (**Figure 2**). They received the following instructions: “Now you have a chance to find a diamond. There are two fields shown below, green, and yellow. One of them contains a desired diamond. Given what you have just learned, which color would you choose? Please select one card.”

Choices satisfy Bayes’ rule, when they are consistent with comparisons of the two Bayesian estimates shown above. We considered four strategies: Bayesian, pre-Bayesian, evidence-only and representativeness (Zhu and Gigerenzer, 2006). One binary choice would not allow us to discern between all the four strategies, as it has two alternatives only: two or more strategies could result in the same choice. Hence, strategies were inferred from pairs of adjacent adventures. To detect strategy use, we looked at eight pairs of adjacent adventures: 1 and 2, 3 and 4, 5 and 6, etc., up to 15 and 16. The second (even-numbered) task in a pair was determined by the first choice so as to allow distinct identification of the strategies used. For example, let us consider the first adventure, specified as (*d, e, f, g*) = (2, 1, 3, 1). This means that we have the following cards: green-diamond (2), yellow-diamond (1), green-stone (3), yellow-stone (1). If a person used representative or evidence-only strategies they would select a green card. If they took a Bayesian or pre-Bayesian approach they would choose yellow. Suppose that a participant selected a yellow card in the first task. The second task in the pair was then specially matched to distinguish between use of a Bayesian or pre-Bayesian strategy. For example, it could take the form of a task specified as (*d, e, f, g*) = (1, 2, 2, 2). If the participant chose a yellow card here, it was concluded that they used a Bayesian strategy. Similarly, other strategies were identified through matching the second task to the choice that was made in the first task in a pair. We used the following patterns of frequencies (*d, e, f, g*): P1 = (2,1,3,1), P2 = (1,2,1,3), P3 = (2,1,2,2), P4 = (2,1,1,3), P5 = (1,2,3,1), and P6 = (3,1,2,1). These served to construct the eight pairs of adventures as follows: pair I (P1 and: P3 when a yellow card was chosen in adventure P1 or P4 when a green card was chosen in P1), pair II (P1 and: P3 or P5), pair III (P1 and: P6 or P4), pair IV (P1 and: P6 or P5), pair V (P2 and: P3 or P4), pair VI (P2 and: P3 or P5), pair VII (P2 and: P6 or P4), and pair VIII (P2 and: P6 or P5). The program randomized the pairs and the on screen allocation of precious and invaluable items on different backgrounds. The content of adventures was also randomized and these consisted of one of the following two stimulus sets: (1) diamonds vs. stones in adventures 1–4 and 9–12, and ambers vs. pieces of glass in adventures 5-8 and 13–16, or (2) diamonds vs. stones in adventures 5–8 and 13–16, and ambers vs. pieces of glass in adventures 1–4 and 9–12.

Because we were looking for consistent application of the four strategies in eight pairs, scores for making choices conforming to Bayes’ rule or other strategies ranged from 0 to 8, summing to 8. We assumed that participants used a strategy consistently and that any deviations from this strategy were accidental. However, it was possible that people might have applied different methods when solving different tasks (because of different task contents, practice, cognitive load, etc.). To provoke use of the same way of thinking in all of the tasks, we provided no feedback during testing so that participants would not learn from practice. Thus, we did not suggest to participants which data they should take into consideration. All tasks were homogeneous in terms of content, format, and difficulty. To minimize cognitive load we limited the learning phase to clicking on seven pictures only and required every adventure to be solved separately. We asked participants to complete all of the tasks at once to prevent any change in skills. We attributed all inconsistencies in responses and strategies applied to random noise and errors.

The Studies 1 and 2 experiments were approved by Scientific Research Ethics Committee at the Faculty of Psychology, University of Warsaw, and informed consent was obtained from all subjects.

### Participants and Procedure

A stratified sample of *N* = 60 students aged 20–35 (*M* = 24.58 years, *SD* = 3.16) volunteered for the study. Participants were equally distributed with regard to gender and type of education (humanities and pure sciences). Individual interviews took place at the University of Warsaw and Warsaw University of Technology. The study was presented as a computer game involving gathering precious items. Completing all of the tasks took about 15 min. Participation was anonymous and not rewarded. At the end, participants were informed about their scores. We then acquainted participants with the actual objectives of the study.

### Results and Interpretation

**Table 1** shows how often each strategy was applied.

**Table 1:** Strategies applied for Bayesian problems in Study 1.

Strategies	Descriptive Statistics (*N* = 60)			
	*Min*	*Max*	*M*	SD
Bayesian	0	8	4.58	2.42
Pre-Bayesian	0	4	0.82	1.13
Representativeness	0	7	2.15	2.07
Evidence-only	0	5	0.45	1.06

Choices conforming to Bayes’ rule were more common than they would be at random [*M* = 4.58, SD = 2.42, test value: μ = 2, *t*(59) = 8.27, *p* < 0.001, *d* = 1.067; Scaled JZS Bayes Factor *B* = 5.15 × 10^8^, supporting μ > 2]. Therefore, the weak version of the hypothesis (Hb) was supported. The Bayesian strategy was dominant and was used in slightly more than half of the cases, however, test statistics were non-significant [test value: μ = 4, *t*(59) = 1.87, *p* = 0.067, *d* = 0.241, with a non-decisive Scaled JZS *B* = 1.283]. Thus, the strong version of the hypothesis (Ha) was not supported.

Participants’ choices conformed to Bayes’ rule in a majority of cases (57%, *M* = 4.58 out of 8), showing that the strategy was used more often than by chance. Furthermore, it was more popular than all the other strategies taken together. The weak hypothesis was supported, but the results involving the strong hypothesis were marginally non-significant. However, the natural sampling procedure demanded that participants computed and compared natural frequencies. This makes natural sampling tasks involving choices potentially more intellectually demanding than pure natural frequency problems. One would therefore expect a greater percentage of fallacious answers when natural sampling is used.

While the adopted methodology resembled natural sampling, it obscured the process of inference underlying choices. A decision based on experience has four phases: (1) gathering information (counting objects); (2) building a mental representation (such as classes of objects and their proportions); (3) processing of information using a choice mechanism (comparison of estimates); (4) making a final selection. Only information gathering and the final decision are external, observable events (Camilleri and Newell, 2009). Therefore, as our results might have appeared to be rather optimistic, we decided to replicate Study 1 but asking participants how they solved the problems in more detail.

## Study 2: Replication of Study 1 with Verbal Protocols

The goal of Study 2 was to replicate Study 1 so as to identify strategies applied in Bayesian tasks more directly. We utilized a process tracing method (Baron, 1994, pp. 19–24). The classical process tracing approach specifies that participants should not be requested to justify their decisions (Nisbett and Wilson, 1977; Ericsson and Simon, 1980). However, participants should easily explain their choices, since the contents of tasks included simple notions, numbers, and computations.

### Materials and Methods

Study 2 was intended to generate results comparable to those from Study 1. The study used the same set of computer tasks as Study 1. After completing the tasks, participants were asked to solve an additional Bayesian exercise. This exercise reproduced a computer task, but was conducted using paper cards. The experimenter presented seven cards with diamonds and stones on and then asked a participant to turn over the cards. After they were all turned over, the cards were taken away and two cards were presented: one yellow and one green. Before uncovering one of them, the participant was asked about the method they used to solve the exercise. The experimenter refrained from providing any suggestions or clues as to how to perform the task or make any computations. Thus, the method applied here differed from the “write aloud” protocols used by Gigerenzer and Hoffrage (1995). At the end of the procedure, the experimenter classified the participant’s answer using the coding list presented in **Table 2**. For example, where a participant compared the natural frequencies of differently colored cards to their total number the experimenter registered this as an evidence-only strategy.

**Table 2:** Coding strategies identified in verbal protocols on the paper task.

Verbal explanation	Interpreted as using the strategy
Comparing relative or absolute frequencies of yellow and green diamonds: *d*/*b* vs. *e*/*b* or *d* vs. *e*	Representativeness
Comparing relative or absolute frequencies of yellow and green cards: *d* + *f* vs. *e* + *g* or (*d* + *f*)/*a* vs. (*e* + *g*)/*a*	Evidence-only
Comparing the relationship of the number of cards with diamonds to the number of cards with defined colors: (*d* + *e*)/(*d* + *f*) vs. (*d* + *e*)/(*e* + *g*)	Pre-Bayesian
Comparing empirical probabilities of cards with diamonds among yellow cards with empirical probabilities of cards with diamonds among green cards: *d*/(*d* + *f*) vs. *e*/(*e* + *g*)	Bayesian
Comparing numbers of cards with diamonds and stones: *b* vs. *c*	Conservatism
Other explanations (mixed strategies, guessing, intuition, etc.)	Mixed/guessing/other

### Participants and Procedure

A sample of *N* = 76 students aged 18–31 (*M* = 23.82 years, SD = 2.17) volunteered for the study. Participants were equally distributed into four cells (*n* = 19 each) with regard to gender and type of education. We applied the same procedure as in Study 1 but added the paper task. The experimenter presented the computer-based tasks from Study 1, followed by the additional exercise, individually to each participant.

### Results

#### Bayesian and Other Strategies

The Bayesian strategy was applied significantly more often than would occur randomly [test value: μ = 2, *t*(75) = 7.41, *p* < 0.001, *d* = 0.850; Scaled JZS *B* = 6.33 × 10^7^, supporting μ > 2 – see **Table 3**]. This strategy again dominated, being utilized in more than half of the cases. Nevertheless, the extent to which use of the strategy exceeded half of the cases was non-significant [test value: μ = 4, *t*(75) = 0.745, *p* > 0.10, *d* = 0.850, Scaled JZS *B* = 1.465 was not decisive]. Thus, again there was support for the weak criterion (Hb), but the strong criterion (Ha) went unsupported. Hence, Study 2 replicated the results of Study 1.

**Table 3:** Strategies applied for Bayesian problems in Study 2.

Strategies	Descriptive statistics (*N* = 76)			
	*Min*	*Max*	*M*	SD
Bayesian	0	8	4.22	2.62
Pre-Bayesian	0	4	0.96	1.08
Representativeness	0	8	2.34	2.24
Evidence-only	0	4	0.47	0.92

#### Verbal Protocol vs. Computer-Based Tasks

Participants’ verbal explanations revealed a new, quite frequently used strategy (32% participants in the whole sample: 18 out of 33 who used the Bayesian strategy in computer tasks, and 8 out of 11 who used heuristics).

The new strategy is different from the strategies listed in **Table 2**. This new strategy included comparing the number of yellow (green) cards among diamonds with the yellow (green) cards among stones. Using the notation we adopted, for yellow this would be: *d*/(*d* + *e*) vs. *f*/(*f* + *g*), and for green: *e*/(*d* + *e*) vs. *g*/(*f* + *g*). Using this strategy does not require inverse thinking about conditions and computing P(H|D) when P(D|H) is given. Intriguingly, this new strategy produces choices that are always the same as choices based on using Bayes’ rule. Comparing *d*/(*d* + *e*) with *f*/(*f* + *g*) is equivalent mathematically with comparing *d* × (*f* + *g*) with *f* × (*d* + *e*), and subsequently: (*d* × *g* + *d* × *f*) with (*e* × *f* + *d* × *f*); *d* × *g* with *e* × *f*; (*d* × *g* + *d* × *e*) with (*e* × *f* + *d* × *e*); *d* × (*e* + *g*) with *e* × (*d* + *f*), and finally *d*/(*d* + *f*) with *e*/(*e* + *g*). This last comparison represents the Bayesian strategy.

Most participants (57 out of 76, i.e., 75%) used consistently algorithmic (Bayesian or the new strategy) or fallacious strategies in both the computer and paper card tasks (**Table 4**).

**Table 4:** Dominant strategies in computer tasks vs. strategies used in the paper task in Study 2.

Dominant strategies in computer tasks	Verbal reports in the paper tasks		Total
	Bayesian or the new strategy	Other strategies	
Bayesian strategy	33 (80%)	8 (20%)	41 (100%)
Other strategies	11 (31%)	24 (69%)	35 (100%)
Total	44 (58%)	32 (42%)	76 (100%)

Thirty-three out of 41 participants (80%), whose dominant strategy was the Bayesian strategy in computer tasks, used the Bayesian strategy or the non-inverse strategy in the paper tasks. Twenty-four out of 35 (69%) used other strategies in both types of tasks. Consistency in using dominant strategies in the computer-based tasks and analogous strategies in paper excercises was moderate [χ^2^(1, *N* = 76) = 18.64, *p* < 0.001, φ = 0.495]. Summing up, Study 2 confirmed the results of Study 1, showing that most choices were consistent with Bayes’ rule. However, they were the result of using of not only Bayes’ strategy, but also the new, non-inverse strategy.

## Study 3 (An Analytical Study)

The Bayesian strategy and the new non-inverse strategy identified in Study 2 provide answers that are always correct in terms of Bayes’ rule. However, people may compromise between the effort and time needed to make consistently correct choices and the practical convenience of making fast and frugal choices. In this section, we investigate how often using fallacious strategies (representativeness, evidence-only and pre-Bayesian strategies) leads to the same choices as does using Bayes’ rule. We analyze strategies with regard to (1) different frequencies expressing decision-makers’ natural sampling experiences and (2) different base rates, arbitrarily defined as rare [P(H) ≤ 0.25], frequent [P(H) ≥ 0.75], and medium [0.25 < P(H) < 0.75].

### Method

Let us start with an example. Consider an elementary situation (*d, e, f, g*) = (4, 1, 1, 1), where *d* denotes number of cards with a diamond on its face and a green back, *e* – diamond-yellow, *f* – stone-green, and *g* – stone-yellow, respectively. Using the Bayesian strategy, a person should choose a green card to reveal a diamond, because: *d*/(*d* + *f*) = 4/(4 + 1) > *e*/(*e* + *g*) = 1/(1 + 1). The same answer would result from using the representativeness strategy [*d*/*b* = 4/5 > *e*/*b* = 1/5], or the evidence-only strategy: (*d* + *f*)/*a* = (4 + 1)/7 > (*e* + *g*)/*a* = (1 + 1)/7. The pre-Bayesian strategy would render solutions greater than one for yellow cards, (*d* + *e*)/(*e* + *g*) = (4 + 1)/(1 + 1) = 5/2. In such cases, when the probability estimates exceed one, we consider the strategy inapplicable.

We wanted to understand how often non-Bayesian strategies return results as good as the correct, Bayesian strategy. We generated all combinations of (*d, e, f, g*) for sampling volumes *d* + *e* + *f* + *g* = *a* ranging from 5 to 50, for *d, e, f, g* > 0 (every combination of data and hypotheses was experienced at least once). For example, **Table 5** shows prescriptions for a choice in all twenty possible elementary situations when *a* = 7. Here, D_1_ means reversing a green card and D_2_ means reversing a yellow card. It turned out that if a was 7, then: (1) the representativeness strategy conforms to Bayes’ rule in 60% of situations; (2) evidence-only – in 50%; (3) pre-Bayesian – in 75% (out of situations where the strategy is applicable).

**Table 5:** Conformity of the heuristic strategies to Bayes’ strategy in choice prescription.

*a*	*d*	*e*	*f*	*g*	Bayesian	Representativeness		Evidence-only		Pre-Bayesian	
					Choice	Choice	Conformity	Choice	Conformity	Choice	Conformity
7	1	1	1	4	D_1_	Any	No	D_2_	No	D_1_	Yes
7	1	1	2	3	D_1_	Any	No	D_2_	No	D_1_	Yes
7	1	1	3	2	D_2_	Any	No	D_1_	No	D_2_	Yes
7	1	1	4	1	D_2_	Any	No	D_1_	No	D_2_	Yes
7	1	2	1	3	D_1_	D_2_	No	D_2_	No	n/a	
7	1	2	2	2	D_2_	D_2_	Yes	D_2_	Yes	D_1_	No
7	1	2	3	1	D_2_	D_2_	Yes	D_1_	No	D_2_	Yes
7	1	3	1	2	D_2_	D_2_	Yes	D_2_	Yes	n/a	
7	1	3	2	1	D_2_	D_2_	Yes	D_2_	Yes	n/a	
7	1	4	1	1	D_2_	D_2_	Yes	D_2_	Yes	n/a	
7	2	1	1	3	D_1_	D_1_	Yes	D_2_	No	D_1_	Yes
7	2	1	2	2	D_1_	D_1_	Yes	D_1_	Yes	D_2_	No
7	2	1	3	1	D_2_	D_1_	No	D_1_	No	n/a	
7	2	2	1	2	D_1_	Any	No	D_2_	no	n/a	
7	2	2	2	1	D_2_	Any	No	D_1_	no	n/a	
7	2	3	1	1	D_2_	D_2_	Yes	D_2_	Yes	n/a	
7	3	1	1	2	D_1_	D_1_	Yes	D_1_	Yes	n/a	
7	3	1	2	1	D_1_	D_1_	Yes	D_1_	Yes	n/a	
7	3	2	1	1	D_1_	D_1_	Yes	D_1_	Yes	n/a	
7	4	1	1	1	D_1_	D_1_	Yes	D_1_	Yes	n/a	

Conformity:						12/20 = 60%		10/20 = 50%		6/8 = 75%	

### Results and Interpretation

**FIGURE 3:**
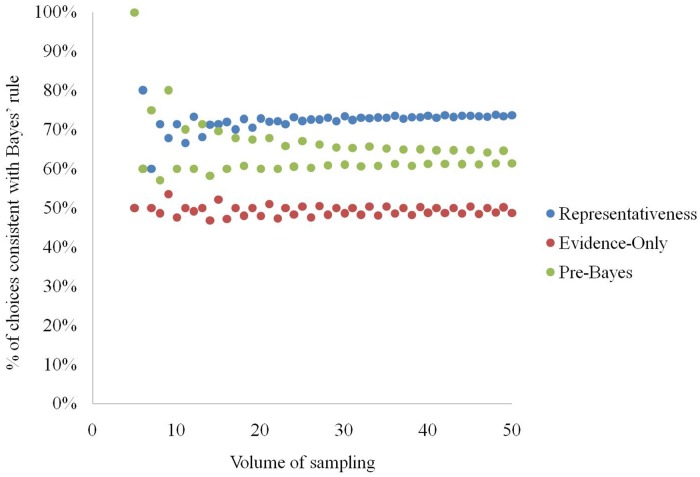
**Natural sampling volume and percentage of elementary situations in which the strategies conform to Bayes’ rule in producing choices**.

The analysis showed that the higher the volume of sampling *a*, the more stable is the percentage of elementary situations in which using a given strategy leads to choices conforming to Bayes rule (see **Figure 3**). The average number of Bayesian solutions returned by a strategy is: (a) representativeness – 73%, (b) evidence-only – 50%, (c) pre-Bayesian – 63%.

**FIGURE 4:**
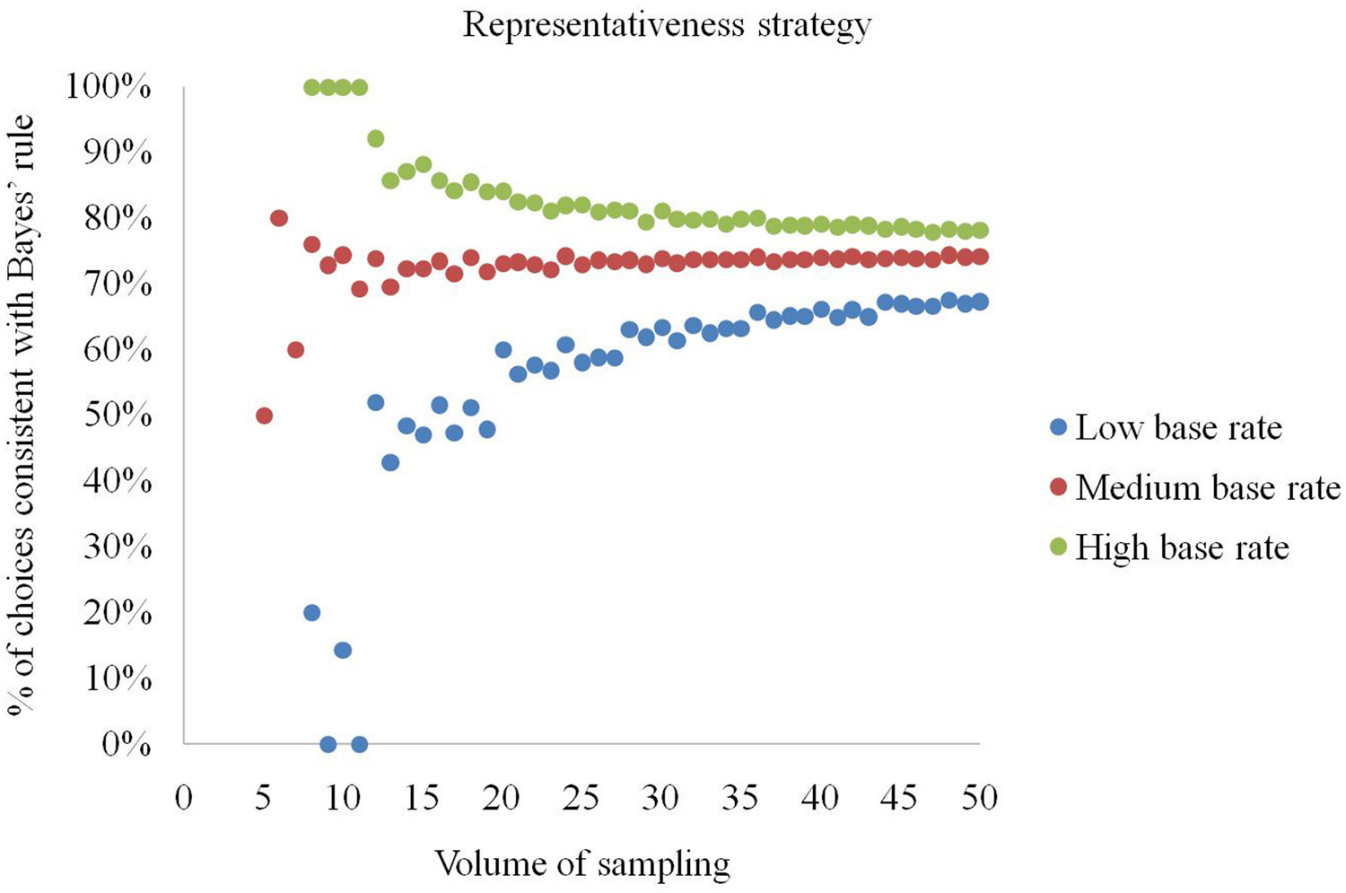
**Percentage of elementary situations in which the representativeness strategy produces choices consistent with Bayes’ rule at low, medium and high base rates**.

The representativeness strategy is effective for high base rates and small natural sampling sizes (**Figure 4**). Specifically, when *a* ≤ 11 and the base rate is *b*/*a* = (*d* + *e*)/(*d* + *e* + *f* + *g*) ≥ 0.75, the representativeness strategy always produces choices conforming to Bayes’ rule. If the base rate exceeds 0.75, the representativeness strategy returns correct choices in no less than 77.9% of cases. However, if the base rate is low (*b*/*a* ≤ 0.25), even if the size is high (*a* > 11), choices conforming to Bayes’ rule are generated at a rate between 42.9% and 67.6%. In contrast, at a low volume of sampling (*a* ≤ 11) and low base rate (*b*/*a* ≤ 0.25) it produces optimal selections in only 20% or fewer situations.

**FIGURE 5:**
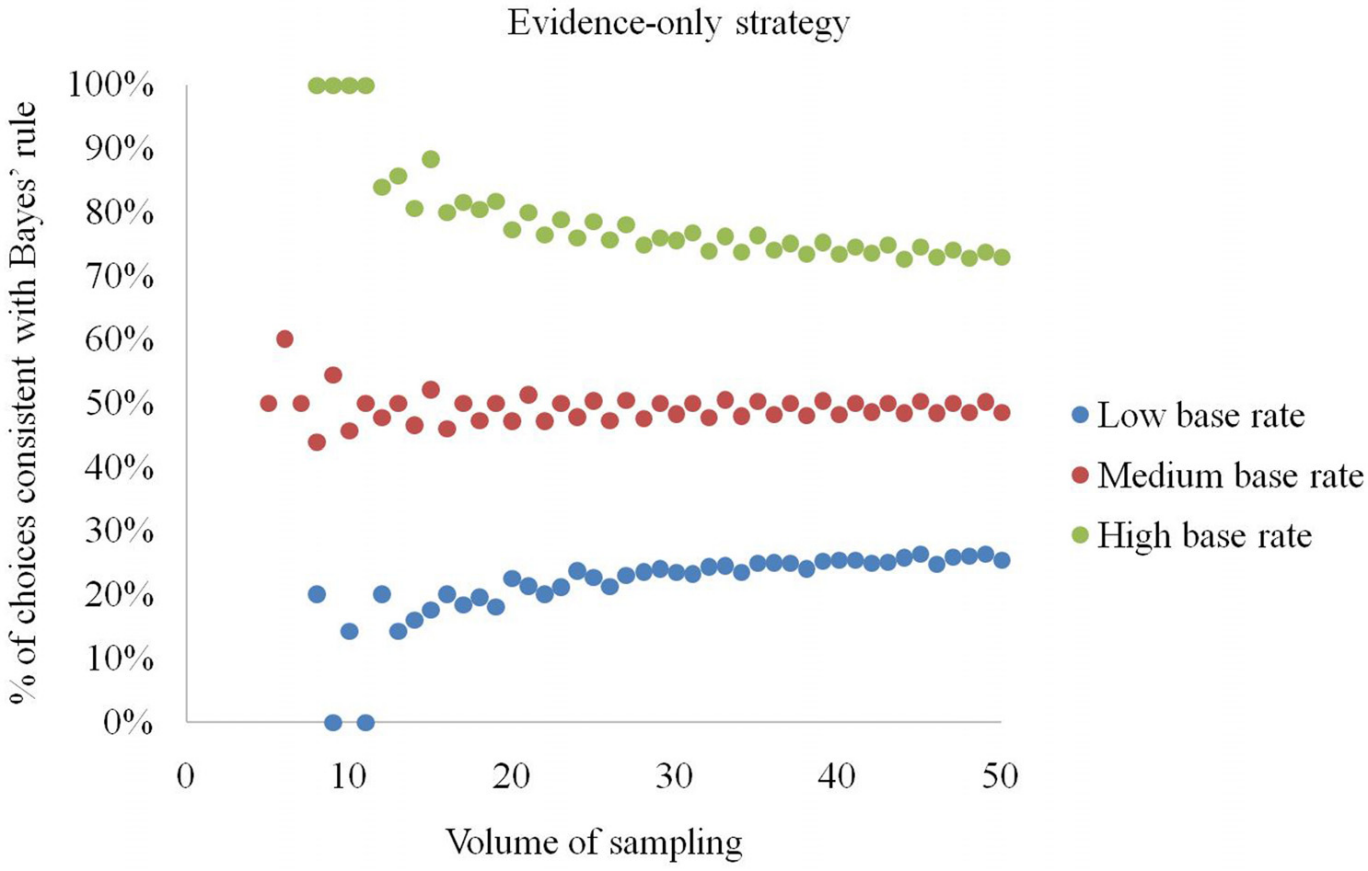
**Percentage of elementary situations in which the evidence-only strategy produces choices consistent with Bayes’ rule at low, medium and high base rates**.

The evidence-only strategy returns choices conforming to Bayes’ rule in 50% of cases at moderate base rates (**Figure 5**). If the base rate (*b*/*a*) exceeds 0.75, the strategy produces correct answers in 72.6% or more of cases. However, when the base rate is lower than 0.25, it produces choices conforming to Bayes’ rule with a probability of 26.5% or less. We also noticed that if *a* ≤ 11 and *b*/*a* ≥ 0.75 the evidence-only strategy is always right. Conversely, for *b*/*a* ≤ 0.25 it renders correct answers in 20% or fewer situations.

**FIGURE 6:**
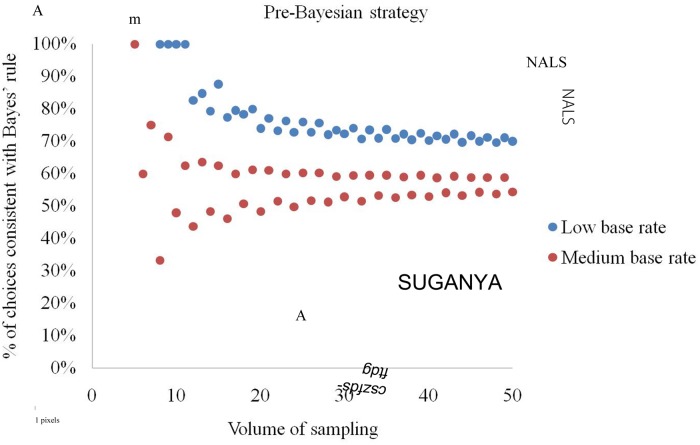
**Percentage of elementary situations in which the pre-Bayesian strategy produces choices consistent with Bayes’ rule at low and medium base rates**.

By definition, the pre-Bayesian strategy always gives opposite answers to the evidence-only strategy (**Figure 6**) and, indeed, we observed its diametrically opposite behavior for all size – base rate combinations. A decision maker should understand that probabilities do not exceed one, i.e., (*d* + *e*)/(*d* + *f*) ≤ 1 and (*d* + *e*)/(*e* + *g*) ≤ 1. This implies 2(*d* + *e*) ≤ (*d* + *f* + *e* + *g*), 2*b* ≤*a* and *b*/*a* ≤ 0.5, and means that the strategy is not applicable for base rates exceeding 1/2. With these assumptions, the strategy renders choices conforming to Bayes’ rule with a probability of 56.0% for medium base rates, and 72.6% for low base rates.

Summing up, the representativeness and evidence-only strategies return choices conforming to Bayes’ rule with very high probabilities if base rates are high and the natural sampling size is low. The pre-Bayesian strategy turned out to be far less efficient.

## Discussion

The first goal of our studies was to find out how often choices in elementary situations satisfy Bayes’ rule, if probabilistic information is acquired through natural sampling. Many studies on Bayesian reasoning have expected that solitary probability estimation should follow the rule. We extended this expectation to choices, however, we did not require participants to evaluate chances, we only asked them make choices.

Our studies confirmed that most choices satisfied Bayes’ rule. Overall, the results were consistent with studies in which the application of natural frequency formats has improved the proportion of Bayesian responses, varying in the range from 31 to 72% (as compared by Barbey and Sloman, 2007), or as high as 77% (in the group of adults investigated by Zhu and Gigerenzer, 2006). One could then conclude that natural sampling facilitates Bayesian inference in elementary situations. Participants were allowed to uncover cards at their own pace and using their own sequences. They discovered connections between objects and colors on their own terms. As we gave no suggestions about how to solve the problems, participants could utilize their own estimates or impressions. Moreover, participants operated on cards at both stages of the task. This compatibility between presented data and answer format could also have enhanced performance (as concluded by Ayal and Beyth-Marom, 2014). Because these results seemed rather optimistic with regard to tasks’ complexity, so we decided to replicate the study adding verbal protocols, which revealed the strategies used more directly.

Although Study 2 replicated the results of Study 1, it turned out that a considerable number of correct choices resulted not from using Bayes’ rule but from a new non-inverse strategy. This method always renders the same answers as the Bayesian strategy in elementary situations and was therefore indistinguishable if only choices were examined. The non-inverse strategy involves computing likelihood ratios, P(D|H) and P(D|not-H), instead of Bayesian posterior probabilities, P(H|D_1_) and P(H|D_2_). In other words, the strategy focuses on a given datum (e.g., the green back of a card) and determines whether it is more characteristic for the hypothesis, H (e.g., a diamond), or for the alternative hypothesis, not-H (a stone). Usually, sticking to likelihood ratios or confusing them with posterior probabilities in Bayesian problems is considered fallacious and is called “an inverse fallacy” (Villejoubert and Mandel, 2002; Mandel, 2014). The confusion of conditions is indeed erroneous, e.g., believing that if most amber is found on yellow beaches then you can find amber on a majority of yellow beaches. However, replacement of both P(H|D_1_) and P(H|D_2_), with both P(D_1_|H) and P(D_1_|not-H), or both P(D_2_|H) and P(D_2_|not-H) is not fallacious. Here, resulting choices are always consistent with Bayes’ rule. The non-inverse strategy is mathematically equivalent to the calculation of the difference P(D|H)–P(D|not-H). This computation was observed in studies by Gigerenzer and Hoffrage (1995), who named it a likelihood subtraction method. These authors concluded that users of this strategy neglect base rate information. However, this might be true only when likelihood ratios are input data, as is the case in typical Bayesian tasks. When natural sampling is applied, as in our studies, people must consider base frequencies for estimating likelihood ratios on their own. This finding supports the proposition that learning from direct experience reduces base-rate neglect (Koehler, 1996; Hertwig and Ortmann, 2001).

Study 3 showed that non-Bayesian, heuristic strategies handled tasks quite well in elementary situations under certain specific circumstances. At low base rates, the pre-Bayesian strategy suggested choices that satisfy Bayes’ rule in most cases at a low volume of natural sampling. The representativeness and evidence-only strategies turned out to be successful under the specific conditions of high base-rates of the distinct hypothesis and low natural sampling sizes (few cards). These findings may explain some difficulties and fallacious propensities in solving Bayesian tasks described in the literature. What would happen, for instance, in the taxi cab problem if, instead of asking participants to give a probability that the taxi cab was blue, we asked them for the probability that the taxi cab was green, given that the witness claimed this to be the case? The findings of such a study would not be very impressive. Fallacious strategies would provide the same interpretation as Bayes’ rule, which would give a 95.8% probability. A conservative strategy would return an estimate of 85%, representativeness – 80%, evidence-only – 71%, and pre-Bayesian – 83.5%. Any strategy would indicate that it was most probably a green cab if a witness claimed it to be so. Thus, it is not necessary to use Bayes’ rule to make a correct decision or judgment based on probability magnitude.

We would like to emphasize that our findings are limited to elementary situations only. Such a limited, local application of strategies and heuristics is consistent with an ecological view. Gigerenzer (1991) pointed out that it is crucial to take into account the environment when one wants to evaluate the approach applied. It is also in line with probabilistic functionalism, which suggests that not using bookish methods for their own sake, but using any methods for achieving goals in the environment, drives human behavior (Pleskac and Hertwig, 2014). The tasks required the selection of green or yellow cards in order to maximize the probability of receiving a diamond instead of a stone.

A natural extension of our studies would be to investigate larger natural sampling sizes and exercises involving more data and more hypotheses. In such a situation the non-inverse strategy does not generalize and would be misleading. Also, heuristic strategies would be likely to be far less efficient in such complex, non-elementary situations.

We are quite pessimistic about humans’ ability to solve such complex problems in a Bayesian way. First, people reveal little interest in gathering complete information on probabilities in naturalistic risky tasks (Huber et al., 1997; Tyszka and Zaleśkiewicz, 2006). Second, if the sample size were increased, working memory boundaries would be exceeded (Anderson, 2000). Longer sampling sequences would probably increase computational complexity, decrease participants’ performance, and provoke them to make more use of various heuristics. The assumption that people use a given strategy consistently within a set of tasks (or at least within pairs of tasks) is challenging and difficult to maintain. This assumption was the main limitation of our studies, but it was necessary to infer strategies from choices indirectly. We tried to minimize the risk of participants using various strategies by presenting only seven cards in a task with homogeneous contents, and giving no feedback. On the one hand, if the assumption is rejected, the problem remains as to how to reveal thinking underlying choices directly, and – at the same time – not to tell participants which chances should be evaluated and how. On the other hand, the assumption is problematic because factors such as skills, cognitive load, learning effects, more differentiated contents, etc. would likely entail applying different heuristics, particularly in more complex tasks.

In analyzing choices in elementary situations we adopted a narrow definition of Bayesian inference as choices or probability evaluations conforming to Bayes’ rule (similarly to other psychological studies investigating Bayesian reasoning). However, Bayesian inference might be understood as the general process of using new information to revise evaluations of likelihoods of events with known prior base rates (Brase and Hill, 2015). In particular, this describes Bayesian analysis of decision problems incorporated in subjective expected utility theory (SEUT, Savage, 1954; Giocoli, 2013; Karni, 2013). According to this perspective, a Bayesian decision-maker’s subjective beliefs are expressed with probabilities which are updated in line with Bayes rule as new information is gathered. Hypothesized outcomes (e.g., diamonds and stones in our studies) are characterized by their utilities [e.g., U(H_1_), U(H_2_), U(H_1_) > U(H_2_)]. The decision maker maximizes the subjective expected utility (SEU) of choice options, combining the subjective probabilities and utilities of outcomes. If the choices are made in elementary situations, as in our studies, maximizing SEU reduces to choosing the option characterized by the higher posterior chance [SEU(D_1_) > SEU(D_2_) when P(H_1_|D_1_) × U(H_1_)+P(H_2_|D_1_) × U(H_2_) > P(H_1_|D_2_) × U(H_1_) + P(H_2_|D_2_) × U(H_2_)], and [P(H_1_|D_1_) – P(H_1_|D_2_)] × [U(H_1_) – U(H_2_)] > 0, and subsequently [P(H_1_|D_1_) – P(H_1_|D_2_) > 0]. However, extending the analysis of choice to more complex situations with more than two hypothesized outcomes (e.g., diamonds, stones, and graphite) entails incorporation of their utilities into the analysis. Here, choice does not reduce to comparing probabilities, and differences among utilities influence the final choice, which is made by maximizing SEU.

Summing up, people performed well in the Bayesian exercises involving natural sampling in elementary situations in our studies. However, correct Bayesian choices can result from using non-Bayesian methods, such as the non-inverse strategy identified in our studies. What is more, even fallacious heuristics produce satisficing choices reasonably often under specific circumstances. Hence, Bayesian inference turns out to be unnecessary in making choices satisfying Bayes’ rule in elementary situations.

## Conflict of Interest Statement

The authors declare that the research was conducted in the absence of any commercial or financial relationships that could be construed as a potential conflict of interest.
